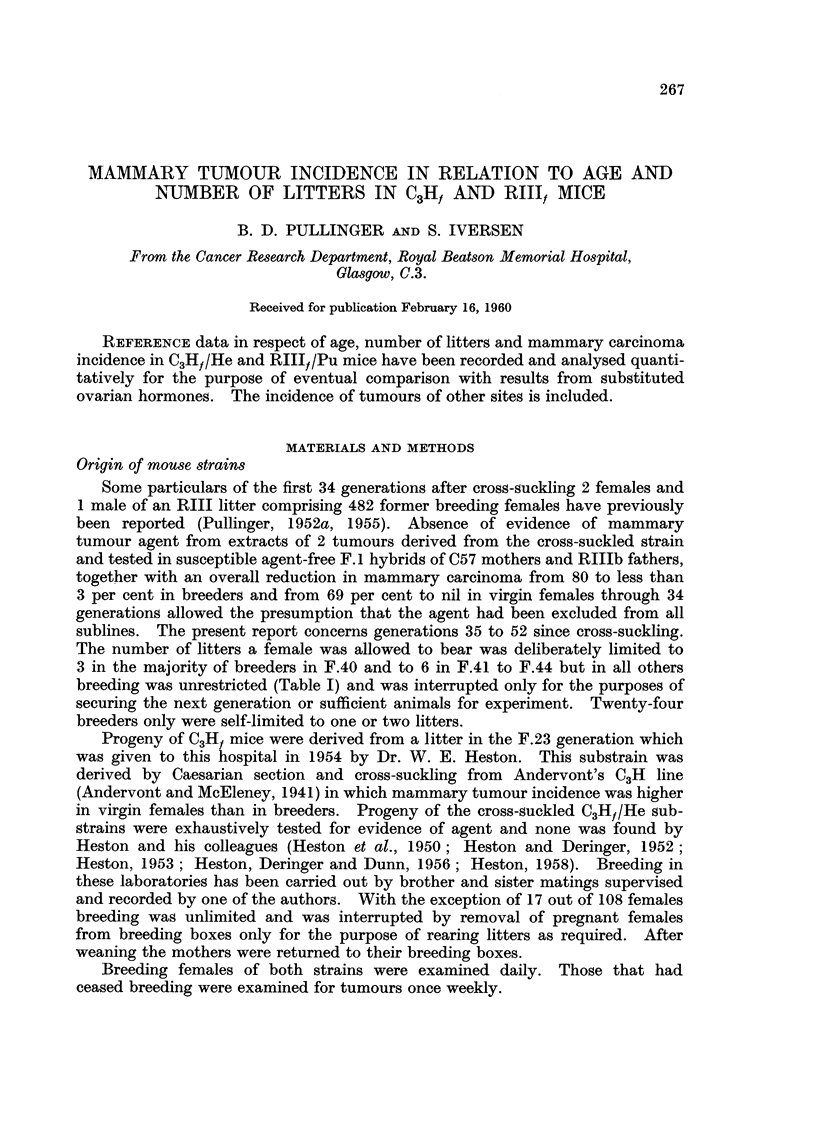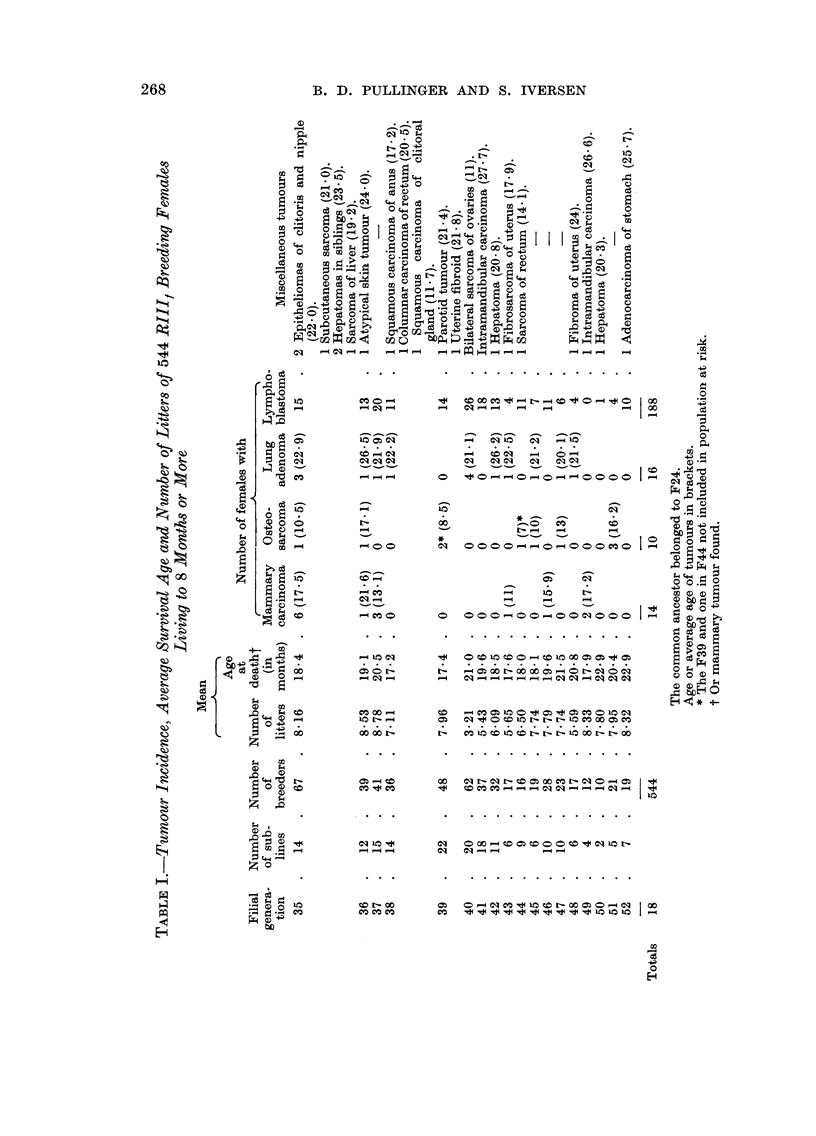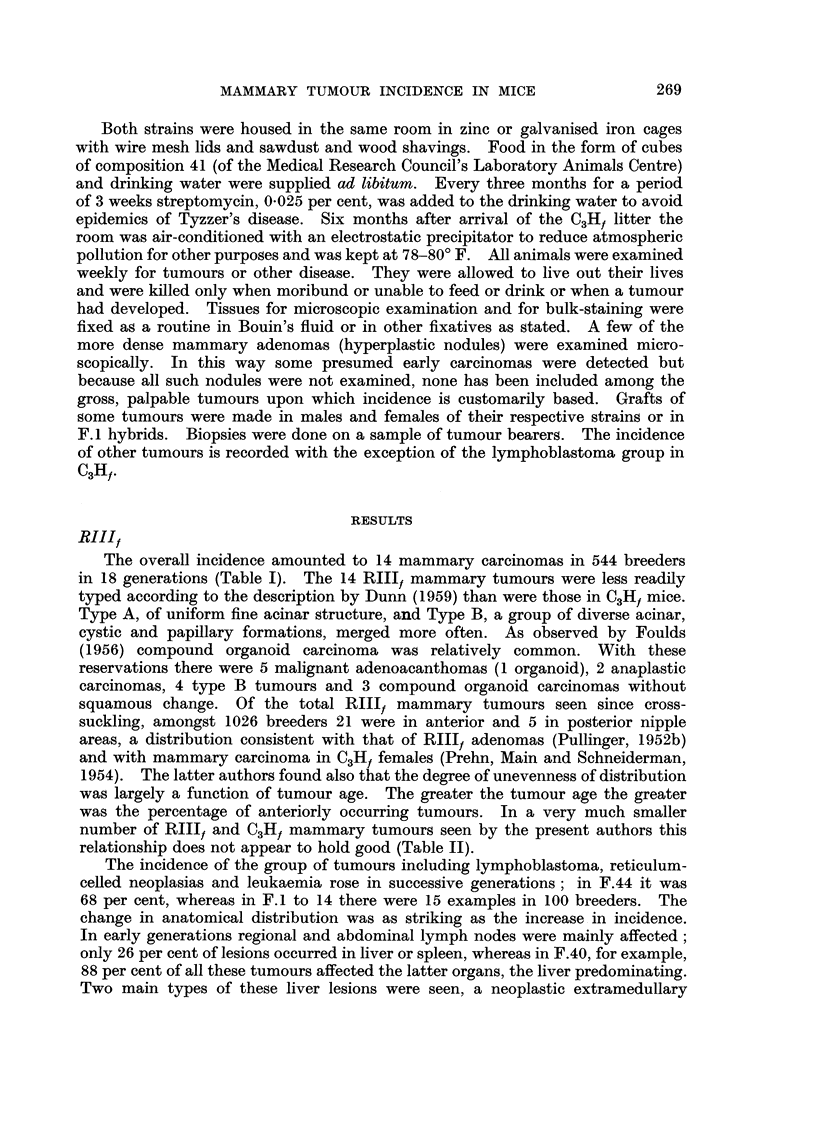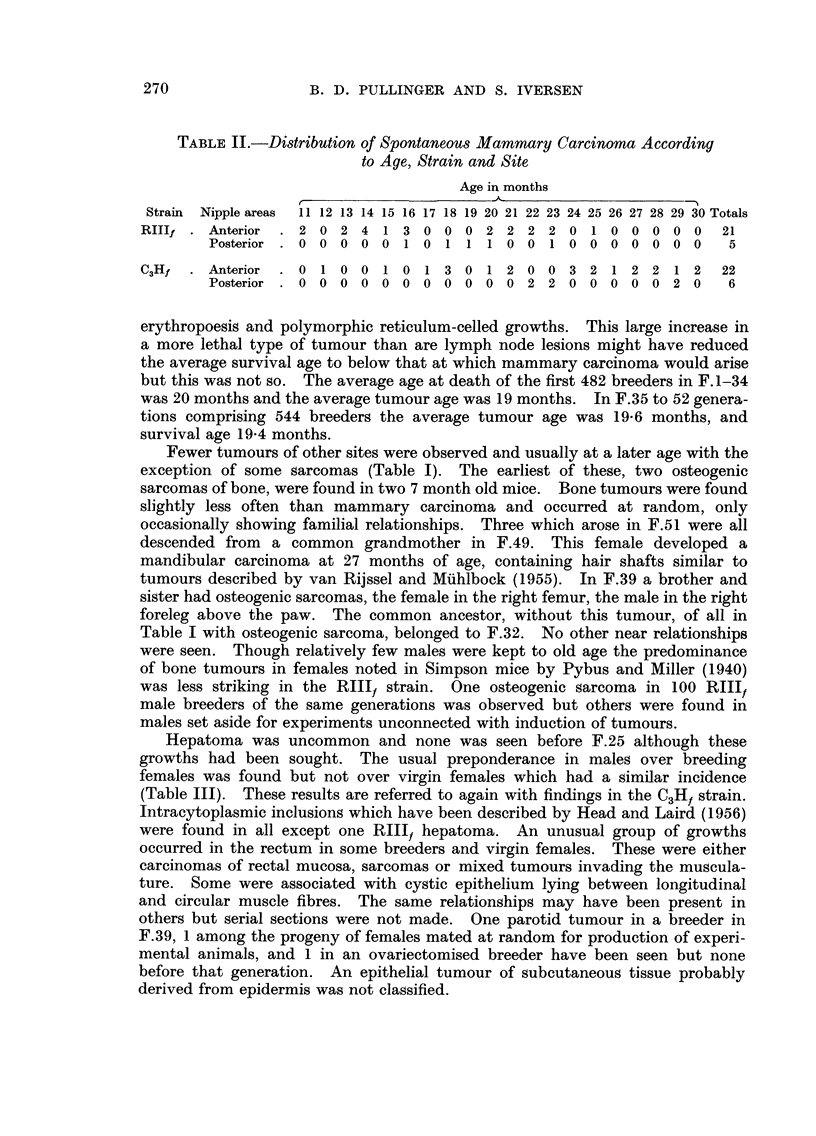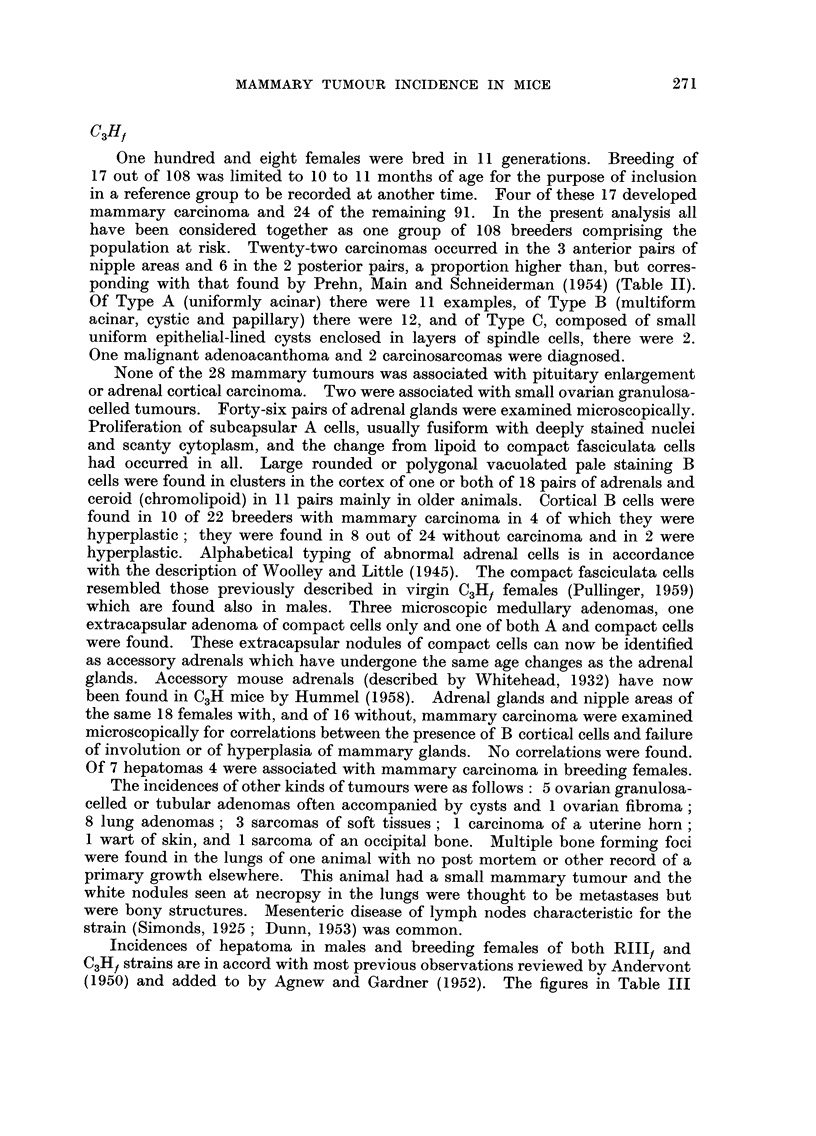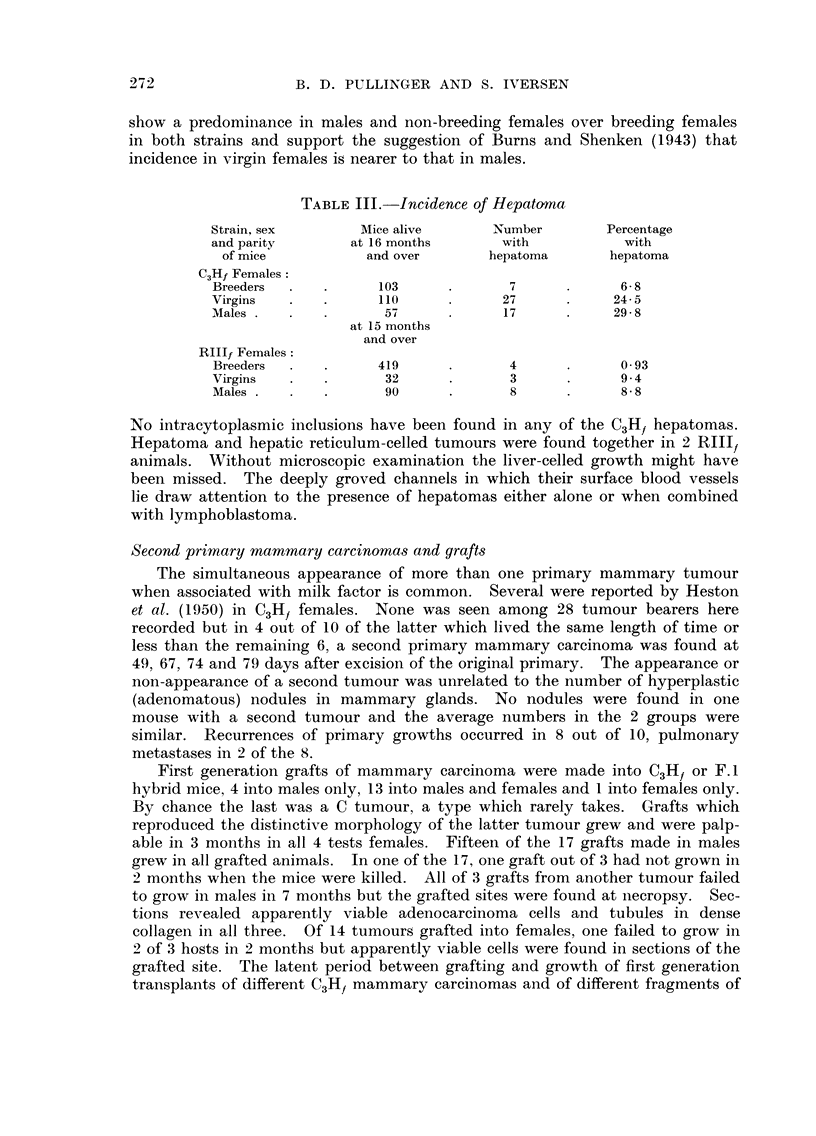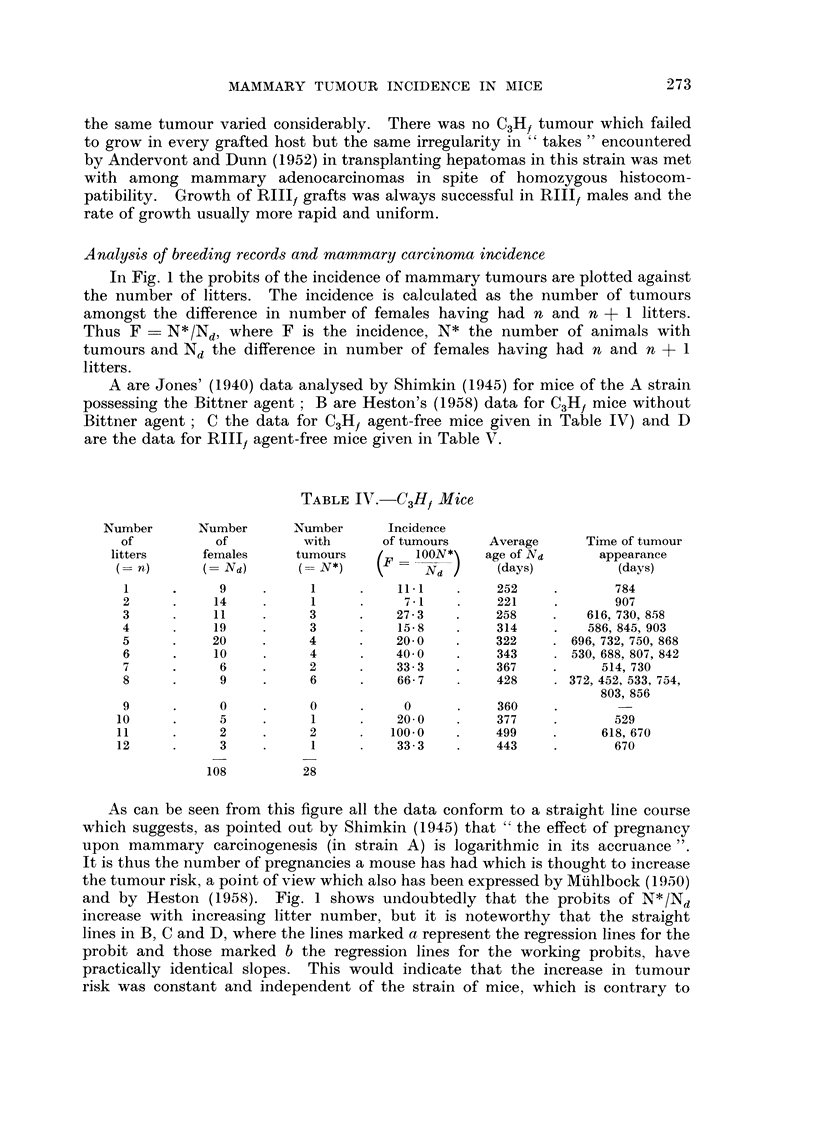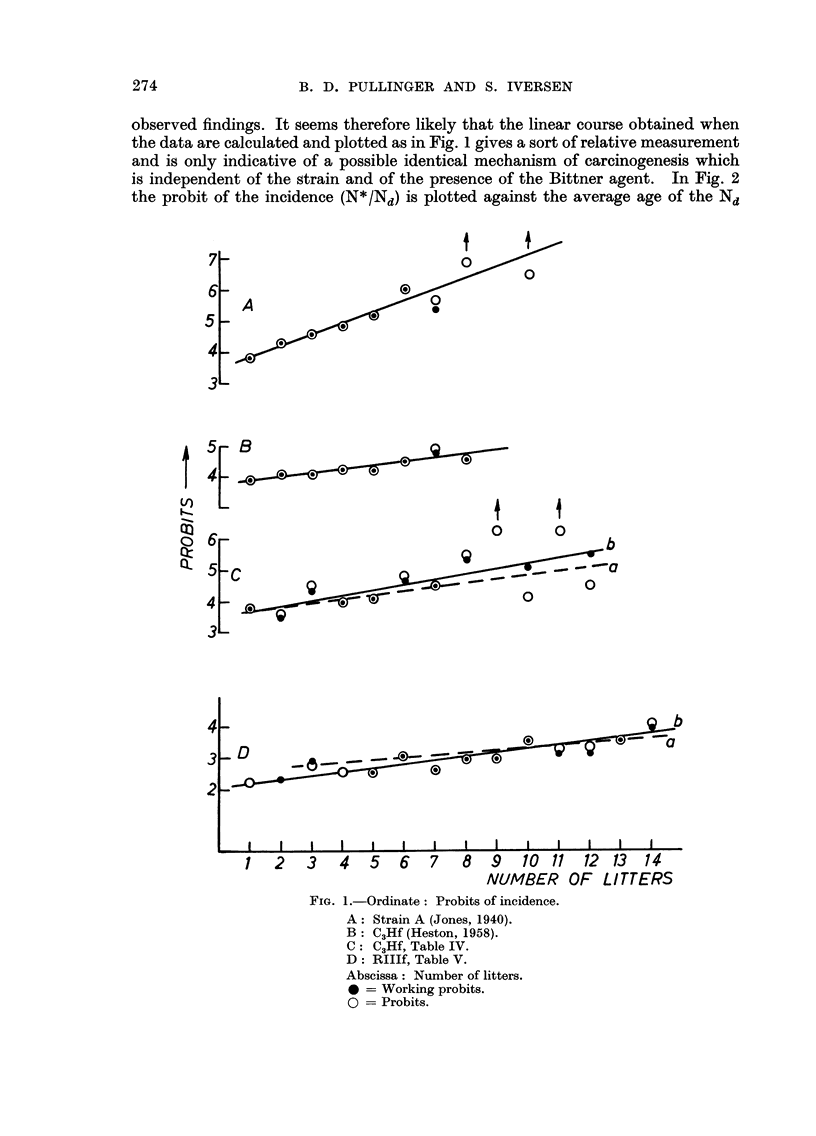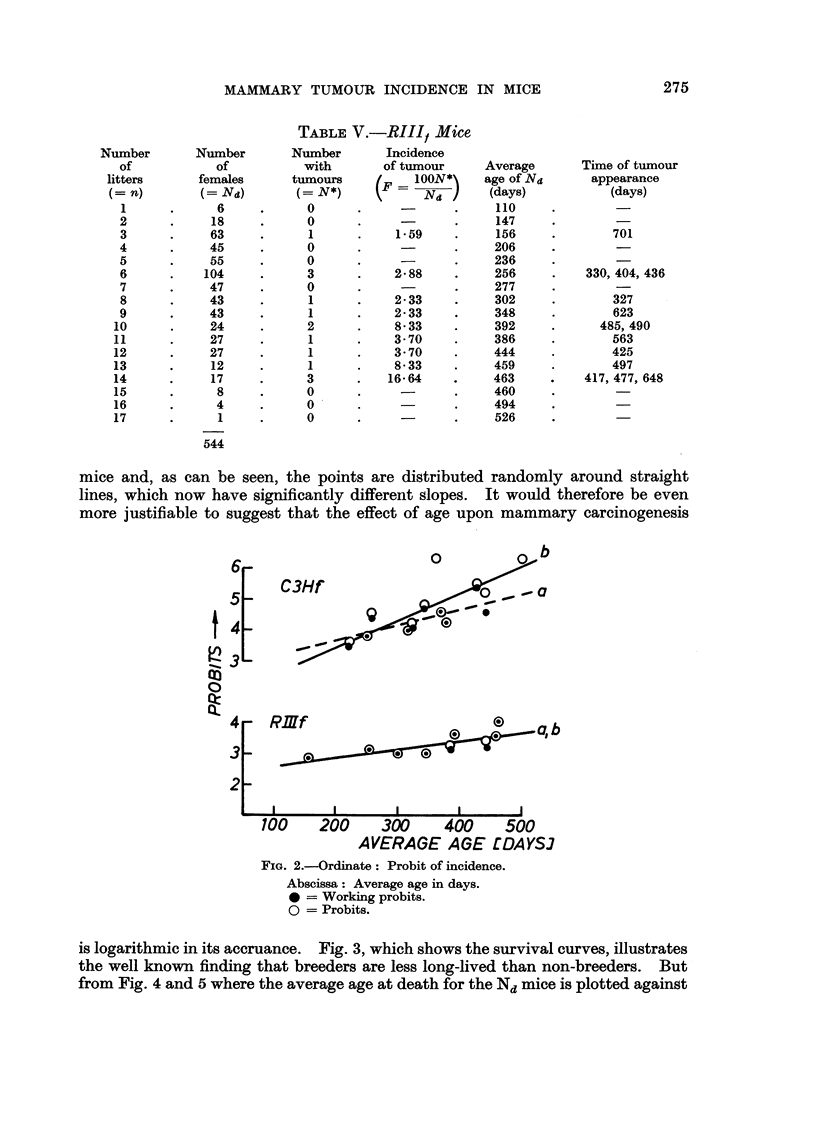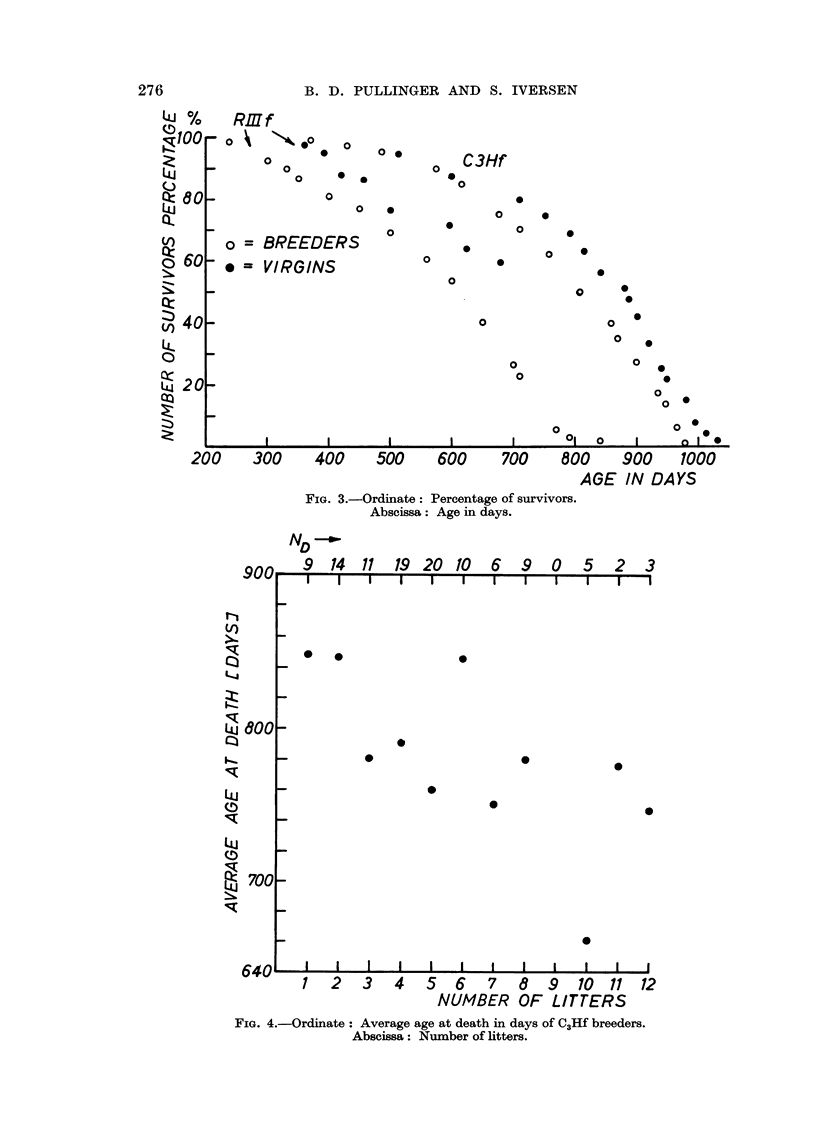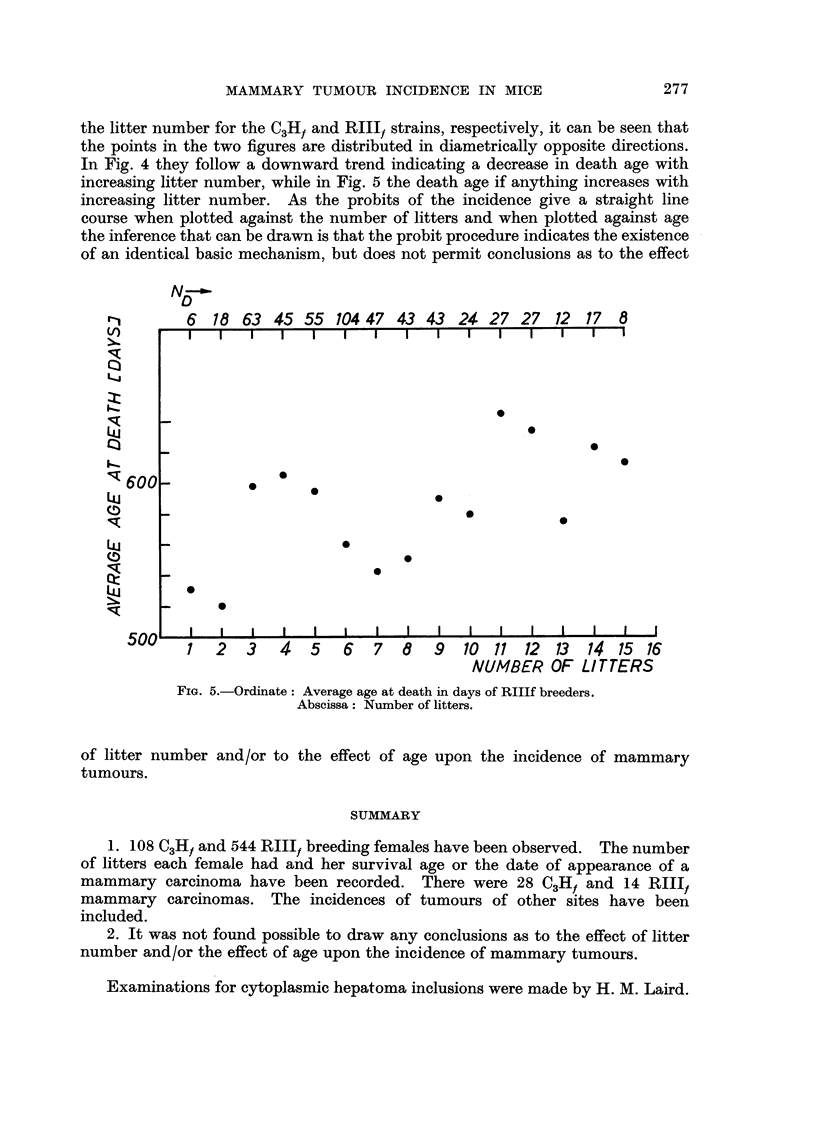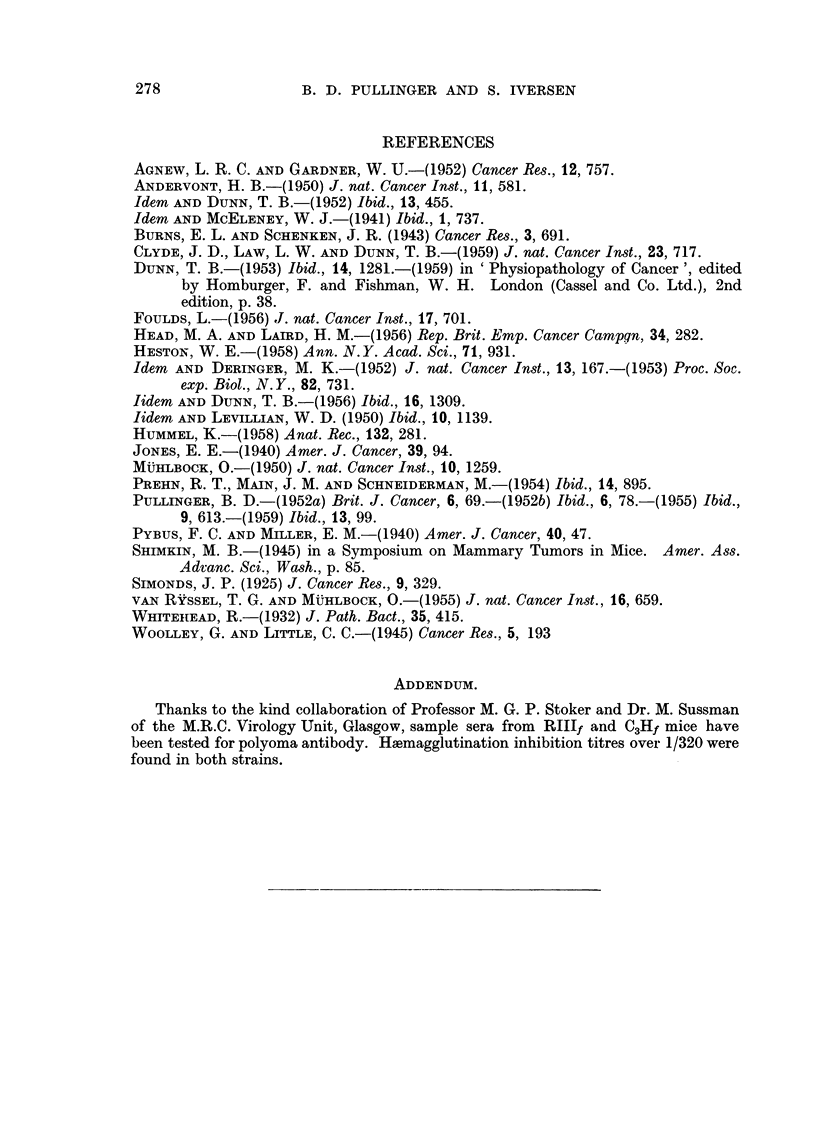# Mammary Tumour Incidence in Relation to Age and Number of Litters in C_3_H_f_ and RIII_f_ Mice

**DOI:** 10.1038/bjc.1960.31

**Published:** 1960-06

**Authors:** B. D. Pullinger, S. Iversen


### MAMMARY TUMOUR INCIDENCE IN RELATION TO AGE AND NUMBER OF LITTERS IN $C_3H_f$ AND $RIII_f$ MICE

B. D. PULLINGER AND S. IVERSEN

*From the Cancer Research Department, Royal Beatson Memorial Hospital, Glasgow, C.3.*

Received for publication February 16, 1960

REFERENCE data in respect of age, number of litters and mammary carcinoma incidence in $C_3H_f$/He and $RIII_f$/Pu mice have been recorded and analysed quantitatively for the purpose of eventual comparison with results from substituted ovarian hormones. The incidence of tumours of other sites is included.

### MATERIALS AND METHODS

### *Origin of mouse strains*

Some particulars of the first 34 generations after cross-suckling 2 females and 1 male of an RIII litter comprising 482 former breeding females have previously been reported (Pullinger, 1952*a*, 1955). Absence of evidence of mammary tumour agent from extracts of 2 tumours derived from the cross-suckled strain and tested in susceptible agent-free F.1 hybrids of C57 mothers and RIIIb fathers, together with an overall reduction in mammary carcinoma from 80 to less than 3 per cent in breeders and from 69 per cent to nil in virgin females through 34 generations allowed the presumption that the agent had been excluded from all sublines. The present report concerns generations 35 to 52 since cross-suckling. The number of litters a female was allowed to bear was deliberately limited to 3 in the majority of breeders in F.40 and to 6 in F.41 to F.44 but in all others breeding was unrestricted (Table I) and was interrupted only for the purposes of securing the next generation or sufficient animals for experiment. Twenty-four breeders only were self-limited to one or two litters.

Progeny of $C_3H_f$ mice were derived from a litter in the F.23 generation which was given to this hospital in 1954 by Dr. W. E. Heston. This substrain was derived by Caesarian section and cross-suckling from Andervont's $C_3H$ line (Andervont and McEleney, 1941) in which mammary tumour incidence was higher in virgin females than in breeders. Progeny of the cross-suckled $C_3H_f$/He substrains were exhaustively tested for evidence of agent and none was found by Heston and his colleagues (Heston *et al.*, 1950 ; Heston and Deringer, 1952 ; Heston, 1953 ; Heston, Deringer and Dunn, 1956 ; Heston, 1958). Breeding in these laboratories has been carried out by brother and sister matings supervised and recorded by one of the authors. With the exception of 17 out of 108 females breeding was unlimited and was interrupted by removal of pregnant females from breeding boxes only for the purpose of rearing litters as required. After weaning the mothers were returned to their breeding boxes.

Breeding females of both strains were examined daily. Those that had ceased breeding were examined for tumours once weekly.

TABLE I.—*Tumour Incidence, Average Survival Age and Number of Litters of 544 $RIII_f$ Breeding Females Living to 8 Months or More*

| | Filial generation | Number of sub-lines | Number of breeders | Mean: Number of litters | Mean: Age at death† (in months) | Number of females with: Mammary carcinoma | Osteo-sarcoma | Lung adenoma | Lympho-blastoma | Miscellaneous tumours |
|---|---|---|---|---|---|---|---|---|---|---|
| | 35 | 14 | 67 | 8·16 | 18·4 | 6 (17·5) | 1 (10·5) | 3 (22·9) | 15 | 2 Epitheliomas of clitoris and nipple (22·0). 1 Subcutaneous sarcoma (21·0). 2 Hepatomas in siblings (23·5). 1 Sarcoma of liver (19·2). |
| | 36 | 12 | 39 | 8·53 | 19·1 | 1 (21·6) | 1 (17·1) | 1 (26·5) | 13 | 1 Atypical skin tumour (24·0). |
| | 37 | 15 | 41 | 8·78 | 20·5 | 3 (13·1) | 0 | 1 (21·9) | 20 | — |
| | 38 | 14 | 36 | 7·11 | 17·2 | 0 | 0 | 1 (22·2) | 11 | 1 Squamous carcinoma of anus (17·2). 1 Columnar carcinoma of rectum (20·5). 1 Squamous carcinoma of clitoral gland (11·7). |
| | 39 | 22 | 48 | 7·96 | 17·4 | 0 | 2* (8·5) | 0 | 14 | 1 Parotid tumour (21·4). 1 Uterine fibroid (21·8). |
| | 40 | 20 | 62 | 3·21 | 21·0 | 0 | 0 | 4 (21·1) | 26 | Bilateral sarcoma of ovaries (11). |
| | 41 | 18 | 37 | 5·43 | 19·6 | 0 | 0 | 0 | 18 | Intramandibular carcinoma (27·7). |
| | 42 | 11 | 32 | 6·09 | 18·5 | 0 | 0 | 1 (26·2) | 13 | 1 Hepatoma (20·8). |
| | 43 | 6 | 17 | 5·65 | 17·6 | 1 (11) | 0 | 1 (22·5) | 4 | 1 Fibrosarcoma of uterus (17·9). |
| | 44 | 9 | 16 | 6·50 | 18·0 | 0 | 1 (7)* | 0 | 11 | 1 Sarcoma of rectum (14·1). |
| | 45 | 6 | 19 | 7·74 | 18·1 | 0 | 1 (10) | 1 (21·2) | 7 | — |
| | 46 | 10 | 28 | 7·79 | 19·6 | 1 (15·9) | 0 | 0 | 11 | — |
| | 47 | 10 | 23 | 7·74 | 21·5 | 0 | 1 (13) | 1 (20·1) | 6 | — |
| | 48 | 6 | 17 | 5·59 | 20·8 | 0 | 0 | 1 (21·5) | 4 | 1 Fibroma of uterus (24). |
| | 49 | 4 | 12 | 8·33 | 17·9 | 2 (17·2) | 0 | 0 | 0 | 1 Intramandibular carcinoma (26·6). |
| | 50 | 2 | 10 | 7·80 | 22·9 | 0 | 0 | 0 | 1 | 1 Hepatoma (20·3). |
| | 51 | 5 | 21 | 7·95 | 20·4 | 0 | 3 (16·2) | 0 | 4 | — |
| | 52 | 7 | 19 | 8·32 | 22·9 | 0 | 0 | 0 | 10 | 1 Adenocarcinoma of stomach (25·7). |
| Totals | 18 | | 544 | | | 14 | 10 | 16 | 188 | |

The common ancestor belonged to F24.

Age or average age of tumours in brackets.

* The F39 and one in F44 not included in population at risk.

† Or mammary tumour found.

Both strains were housed in the same room in zinc or galvanised iron cages with wire mesh lids and sawdust and wood shavings. Food in the form of cubes of composition 41 (of the Medical Research Council's Laboratory Animals Centre) and drinking water were supplied *ad libitum*. Every three months for a period of 3 weeks streptomycin, 0·025 per cent, was added to the drinking water to avoid epidemics of Tyzzer's disease. Six months after arrival of the $C_3H_f$ litter the room was air-conditioned with an electrostatic precipitator to reduce atmospheric pollution for other purposes and was kept at 78–80° F. All animals were examined weekly for tumours or other disease. They were allowed to live out their lives and were killed only when moribund or unable to feed or drink or when a tumour had developed. Tissues for microscopic examination and for bulk-staining were fixed as a routine in Bouin's fluid or in other fixatives as stated. A few of the more dense mammary adenomas (hyperplastic nodules) were examined microscopically. In this way some presumed early carcinomas were detected but because all such nodules were not examined, none has been included among the gross, palpable tumours upon which incidence is customarily based. Grafts of some tumours were made in males and females of their respective strains or in F.1 hybrids. Biopsies were done on a sample of tumour bearers. The incidence of other tumours is recorded with the exception of the lymphoblastoma group in $C_3H_f$.

### RESULTS

### *$RIII_f$*

The overall incidence amounted to 14 mammary carcinomas in 544 breeders in 18 generations (Table I). The 14 $RIII_f$ mammary tumours were less readily typed according to the description by Dunn (1959) than were those in $C_3H_f$ mice. Type A, of uniform fine acinar structure, and Type B, a group of diverse acinar, cystic and papillary formations, merged more often. As observed by Foulds (1956) compound organoid carcinoma was relatively common. With these reservations there were 5 malignant adenoacanthomas (1 organoid), 2 anaplastic carcinomas, 4 type B tumours and 3 compound organoid carcinomas without squamous change. Of the total $RIII_f$ mammary tumours seen since cross-suckling, amongst 1026 breeders 21 were in anterior and 5 in posterior nipple areas, a distribution consistent with that of $RIII_f$ adenomas (Pullinger, 1952b) and with mammary carcinoma in $C_3H_f$ females (Prehn, Main and Schneiderman, 1954). The latter authors found also that the degree of unevenness of distribution was largely a function of tumour age. The greater the tumour age the greater was the percentage of anteriorly occurring tumours. In a very much smaller number of $RIII_f$ and $C_3H_f$ mammary tumours seen by the present authors this relationship does not appear to hold good (Table II).

The incidence of the group of tumours including lymphoblastoma, reticulum-celled neoplasias and leukaemia rose in successive generations ; in F.44 it was 68 per cent, whereas in F.1 to 14 there were 15 examples in 100 breeders. The change in anatomical distribution was as striking as the increase in incidence. In early generations regional and abdominal lymph nodes were mainly affected ; only 26 per cent of lesions occurred in liver or spleen, whereas in F.40, for example, 88 per cent of all these tumours affected the latter organs, the liver predominating. Two main types of these liver lesions were seen, a neoplastic extramedullary

TABLE II.—*Distribution of Spontaneous Mammary Carcinoma According to Age, Strain and Site*

| | | Age in months | | | | | | | | | | | | | | | | | | | | |
|---|---|---|---|---|---|---|---|---|---|---|---|---|---|---|---|---|---|---|---|---|---|---|
| Strain | Nipple areas | 11 | 12 | 13 | 14 | 15 | 16 | 17 | 18 | 19 | 20 | 21 | 22 | 23 | 24 | 25 | 26 | 27 | 28 | 29 | 30 | Totals |
| $RIII_f$ | Anterior | 2 | 0 | 2 | 4 | 1 | 3 | 0 | 0 | 0 | 2 | 2 | 2 | 2 | 0 | 1 | 0 | 0 | 0 | 0 | 0 | 21 |
| | Posterior | 0 | 0 | 0 | 0 | 0 | 1 | 0 | 1 | 1 | 1 | 0 | 0 | 1 | 0 | 0 | 0 | 0 | 0 | 0 | 0 | 5 |
| $C_3H_f$ | Anterior | 0 | 1 | 0 | 0 | 1 | 0 | 1 | 3 | 0 | 1 | 2 | 0 | 0 | 3 | 2 | 1 | 2 | 2 | 1 | 2 | 22 |
| | Posterior | 0 | 0 | 0 | 0 | 0 | 0 | 0 | 0 | 0 | 0 | 0 | 2 | 2 | 0 | 0 | 0 | 0 | 0 | 2 | 0 | 6 |

erythropoesis and polymorphic reticulum-celled growths. This large increase in a more lethal type of tumour than are lymph node lesions might have reduced the average survival age to below that at which mammary carcinoma would arise but this was not so. The average age at death of the first 482 breeders in F.1–34 was 20 months and the average tumour age was 19 months. In F.35 to 52 generations comprising 544 breeders the average tumour age was 19·6 months, and survival age 19·4 months.

Fewer tumours of other sites were observed and usually at a later age with the exception of some sarcomas (Table I). The earliest of these, two osteogenic sarcomas of bone, were found in two 7 month old mice. Bone tumours were found slightly less often than mammary carcinoma and occurred at random, only occasionally showing familial relationships. Three which arose in F.51 were all descended from a common grandmother in F.49. This female developed a mandibular carcinoma at 27 months of age, containing hair shafts similar to tumours described by van Rijssel and Mühlbock (1955). In F.39 a brother and sister had osteogenic sarcomas, the female in the right femur, the male in the right foreleg above the paw. The common ancestor, without this tumour, of all in Table I with osteogenic sarcoma, belonged to F.32. No other near relationships were seen. Though relatively few males were kept to old age the predominance of bone tumours in females noted in Simpson mice by Pybus and Miller (1940) was less striking in the $RIII_f$ strain. One osteogenic sarcoma in 100 $RIII_f$ male breeders of the same generations was observed but others were found in males set aside for experiments unconnected with induction of tumours.

Hepatoma was uncommon and none was seen before F.25 although these growths had been sought. The usual preponderance in males over breeding females was found but not over virgin females which had a similar incidence (Table III). These results are referred to again with findings in the $C_3H_f$ strain. Intracytoplasmic inclusions which have been described by Head and Laird (1956) were found in all except one $RIII_f$ hepatoma. An unusual group of growths occurred in the rectum in some breeders and virgin females. These were either carcinomas of rectal mucosa, sarcomas or mixed tumours invading the musculature. Some were associated with cystic epithelium lying between longitudinal and circular muscle fibres. The same relationships may have been present in others but serial sections were not made. One parotid tumour in a breeder in F.39, 1 among the progeny of females mated at random for production of experimental animals, and 1 in an ovariectomised breeder have been seen but none before that generation. An epithelial tumour of subcutaneous tissue probably derived from epidermis was not classified.

$C_3H_f$

One hundred and eight females were bred in 11 generations. Breeding of 17 out of 108 was limited to 10 to 11 months of age for the purpose of inclusion in a reference group to be recorded at another time. Four of these 17 developed mammary carcinoma and 24 of the remaining 91. In the present analysis all have been considered together as one group of 108 breeders comprising the population at risk. Twenty-two carcinomas occurred in the 3 anterior pairs of nipple areas and 6 in the 2 posterior pairs, a proportion higher than, but corresponding with that found by Prehn, Main and Schneiderman (1954) (Table II). Of Type A (uniformly acinar) there were 11 examples, of Type B (multiform acinar, cystic and papillary) there were 12, and of Type C, composed of small uniform epithelial-lined cysts enclosed in layers of spindle cells, there were 2. One malignant adenoacanthoma and 2 carcinosarcomas were diagnosed.

None of the 28 mammary tumours was associated with pituitary enlargement or adrenal cortical carcinoma. Two were associated with small ovarian granulosa-celled tumours. Forty-six pairs of adrenal glands were examined microscopically. Proliferation of subcapsular A cells, usually fusiform with deeply stained nuclei and scanty cytoplasm, and the change from lipoid to compact fasciculata cells had occurred in all. Large rounded or polygonal vacuolated pale staining B cells were found in clusters in the cortex of one or both of 18 pairs of adrenals and ceroid (chromolipoid) in 11 pairs mainly in older animals. Cortical B cells were found in 10 of 22 breeders with mammary carcinoma in 4 of which they were hyperplastic ; they were found in 8 out of 24 without carcinoma and in 2 were hyperplastic. Alphabetical typing of abnormal adrenal cells is in accordance with the description of Woolley and Little (1945). The compact fasciculata cells resembled those previously described in virgin $C_3H_f$ females (Pullinger, 1959) which are found also in males. Three microscopic medullary adenomas, one extracapsular adenoma of compact cells only and one of both A and compact cells were found. These extracapsular nodules of compact cells can now be identified as accessory adrenals which have undergone the same age changes as the adrenal glands. Accessory mouse adrenals (described by Whitehead, 1932) have now been found in $C_3H$ mice by Hummel (1958). Adrenal glands and nipple areas of the same 18 females with, and of 16 without, mammary carcinoma were examined microscopically for correlations between the presence of B cortical cells and failure of involution or of hyperplasia of mammary glands. No correlations were found. Of 7 hepatomas 4 were associated with mammary carcinoma in breeding females.

The incidences of other kinds of tumours were as follows : 5 ovarian granulosa-celled or tubular adenomas often accompanied by cysts and 1 ovarian fibroma ; 8 lung adenomas ; 3 sarcomas of soft tissues ; 1 carcinoma of a uterine horn ; 1 wart of skin, and 1 sarcoma of an occipital bone. Multiple bone forming foci were found in the lungs of one animal with no post mortem or other record of a primary growth elsewhere. This animal had a small mammary tumour and the white nodules seen at necropsy in the lungs were thought to be metastases but were bony structures. Mesenteric disease of lymph nodes characteristic for the strain (Simonds, 1925 ; Dunn, 1953) was common.

Incidences of hepatoma in males and breeding females of both $RIII_f$ and $C_3H_f$ strains are in accord with most previous observations reviewed by Andervont (1950) and added to by Agnew and Gardner (1952). The figures in Table III

show a predominance in males and non-breeding females over breeding females in both strains and support the suggestion of Burns and Shenken (1943) that incidence in virgin females is nearer to that in males.

TABLE III.—*Incidence of Hepatoma*

| Strain, sex and parity of mice | Mice alive at 16 months and over | Number with hepatoma | Percentage with hepatoma |
|---|---|---|---|
| $C_3H_f$ Females : | | | |
| Breeders | 103 | 7 | 6·8 |
| Virgins | 110 | 27 | 24·5 |
| Males | 57 | 17 | 29·8 |
| | at 15 months and over | | |
| $RIII_f$ Females : | | | |
| Breeders | 419 | 4 | 0·93 |
| Virgins | 32 | 3 | 9·4 |
| Males | 90 | 8 | 8·8 |

No intracytoplasmic inclusions have been found in any of the $C_3H_f$ hepatomas. Hepatoma and hepatic reticulum-celled tumours were found together in 2 $RIII_f$ animals. Without microscopic examination the liver-celled growth might have been missed. The deeply groved channels in which their surface blood vessels lie draw attention to the presence of hepatomas either alone or when combined with lymphoblastoma.

*Second primary mammary carcinomas and grafts*

The simultaneous appearance of more than one primary mammary tumour when associated with milk factor is common. Several were reported by Heston *et al.* (1950) in $C_3H_f$ females. None was seen among 28 tumour bearers here recorded but in 4 out of 10 of the latter which lived the same length of time or less than the remaining 6, a second primary mammary carcinoma was found at 49, 67, 74 and 79 days after excision of the original primary. The appearance or non-appearance of a second tumour was unrelated to the number of hyperplastic (adenomatous) nodules in mammary glands. No nodules were found in one mouse with a second tumour and the average numbers in the 2 groups were similar. Recurrences of primary growths occurred in 8 out of 10, pulmonary metastases in 2 of the 8.

First generation grafts of mammary carcinoma were made into $C_3H_f$ or F.1 hybrid mice, 4 into males only, 13 into males and females and 1 into females only. By chance the last was a C tumour, a type which rarely takes. Grafts which reproduced the distinctive morphology of the latter tumour grew and were palpable in 3 months in all 4 tests females. Fifteen of the 17 grafts made in males grew in all grafted animals. In one of the 17, one graft out of 3 had not grown in 2 months when the mice were killed. All of 3 grafts from another tumour failed to grow in males in 7 months but the grafted sites were found at necropsy. Sections revealed apparently viable adenocarcinoma cells and tubules in dense collagen in all three. Of 14 tumours grafted into females, one failed to grow in 2 of 3 hosts in 2 months but apparently viable cells were found in sections of the grafted site. The latent period between grafting and growth of first generation transplants of different $C_3H_f$ mammary carcinomas and of different fragments of the same tumour varied considerably. There was no $C_3H_f$ tumour which failed to grow in every grafted host but the same irregularity in " takes " encountered by Andervont and Dunn (1952) in transplanting hepatomas in this strain was met with among mammary adenocarcinomas in spite of homozygous histocompatibility. Growth of $RIII_f$ grafts was always successful in $RIII_f$ males and the rate of growth usually more rapid and uniform.

*Analysis of breeding records and mammary carcinoma incidence*

In Fig. 1 the probits of the incidence of mammary tumours are plotted against the number of litters. The incidence is calculated as the number of tumours amongst the difference in number of females having had $n$ and $n + 1$ litters. Thus $F = N^*/N_d$, where F is the incidence, N* the number of animals with tumours and $N_d$ the difference in number of females having had $n$ and $n + 1$ litters.

A are Jones' (1940) data analysed by Shimkin (1945) for mice of the A strain possessing the Bittner agent ; B are Heston's (1958) data for $C_3H_f$ mice without Bittner agent ; C the data for $C_3H_f$ agent-free mice given in Table IV) and D are the data for $RIII_f$ agent-free mice given in Table V.

TABLE IV.—$C_3H_f$ *Mice*

| Number of litters ($= n$) | Number of females ($= N_d$) | Number with tumours ($= N^*$) | Incidence of tumours $\left(F = \frac{100N^*}{N_d}\right)$ | Average age of $N_d$ (days) | Time of tumour appearance (days) |
|---|---|---|---|---|---|
| 1 | 9 | 1 | 11·1 | 252 | 784 |
| 2 | 14 | 1 | 7·1 | 221 | 907 |
| 3 | 11 | 3 | 27·3 | 258 | 616, 730, 858 |
| 4 | 19 | 3 | 15·8 | 314 | 586, 845, 903 |
| 5 | 20 | 4 | 20·0 | 322 | 696, 732, 750, 868 |
| 6 | 10 | 4 | 40·0 | 343 | 530, 688, 807, 842 |
| 7 | 6 | 2 | 33·3 | 367 | 514, 730 |
| 8 | 9 | 6 | 66·7 | 428 | 372, 452, 533, 754, 803, 856 |
| 9 | 0 | 0 | 0 | 360 | — |
| 10 | 5 | 1 | 20·0 | 377 | 529 |
| 11 | 2 | 2 | 100·0 | 499 | 618, 670 |
| 12 | 3 | 1 | 33·3 | 443 | 670 |
| | 108 | 28 | | | |

As can be seen from this figure all the data conform to a straight line course which suggests, as pointed out by Shimkin (1945) that " the effect of pregnancy upon mammary carcinogenesis (in strain A) is logarithmic in its accruance ". It is thus the number of pregnancies a mouse has had which is thought to increase the tumour risk, a point of view which also has been expressed by Mühlbock (1950) and by Heston (1958). Fig. 1 shows undoubtedly that the probits of $N^*/N_d$ increase with increasing litter number, but it is noteworthy that the straight lines in B, C and D, where the lines marked $a$ represent the regression lines for the probit and those marked $b$ the regression lines for the working probits, have practically identical slopes. This would indicate that the increase in tumour risk was constant and independent of the strain of mice, which is contrary to observed findings. It seems therefore likely that the linear course obtained when the data are calculated and plotted as in Fig. 1 gives a sort of relative measurement and is only indicative of a possible identical mechanism of carcinogenesis which is independent of the strain and of the presence of the Bittner agent. In Fig. 2 the probit of the incidence ($N^*/N_d$) is plotted against the average age of the $N_d$

FIG. 1.—Ordinate : Probits of incidence.

A : Strain A (Jones, 1940).

B : $C_3Hf$ (Heston, 1958).

C : $C_3Hf$, Table IV.

D : RIIIf, Table V.

Abscissa : Number of litters.

● = Working probits.

○ = Probits.

TABLE V.—*RIII$_f$ Mice*

| Number of litters (= $n$) | Number of females (= $N_d$) | Number with tumours (= $N^*$) | Incidence of tumour $\left(F = \frac{100N^*}{N_d}\right)$ | Average age of $N_d$ (days) | Time of tumour appearance (days) |
|---|---|---|---|---|---|
| 1 | 6 | 0 | — | 110 | — |
| 2 | 18 | 0 | — | 147 | — |
| 3 | 63 | 1 | 1·59 | 156 | 701 |
| 4 | 45 | 0 | — | 206 | — |
| 5 | 55 | 0 | — | 236 | — |
| 6 | 104 | 3 | 2·88 | 256 | 330, 404, 436 |
| 7 | 47 | 0 | — | 277 | — |
| 8 | 43 | 1 | 2·33 | 302 | 327 |
| 9 | 43 | 1 | 2·33 | 348 | 623 |
| 10 | 24 | 2 | 8·33 | 392 | 485, 490 |
| 11 | 27 | 1 | 3·70 | 386 | 563 |
| 12 | 27 | 1 | 3·70 | 444 | 425 |
| 13 | 12 | 1 | 8·33 | 459 | 497 |
| 14 | 17 | 3 | 16·64 | 463 | 417, 477, 648 |
| 15 | 8 | 0 | — | 460 | — |
| 16 | 4 | 0 | — | 494 | — |
| 17 | 1 | 0 | — | 526 | — |
| | 544 | | | | |

mice and, as can be seen, the points are distributed randomly around straight lines, which now have significantly different slopes. It would therefore be even more justifiable to suggest that the effect of age upon mammary carcinogenesis

FIG. 2.—Ordinate : Probit of incidence.

Abscissa : Average age in days.

● = Working probits.

○ = Probits.

is logarithmic in its accruance. Fig. 3, which shows the survival curves, illustrates the well known finding that breeders are less long-lived than non-breeders. But from Fig. 4 and 5 where the average age at death for the $N_d$ mice is plotted against

FIG. 3.—Ordinate : Percentage of survivors.
Abscissa : Age in days.

FIG. 4.—Ordinate : Average age at death in days of $C_3Hf$ breeders.
Abscissa : Number of litters.

the litter number for the $C_3H_f$ and $RIII_f$ strains, respectively, it can be seen that the points in the two figures are distributed in diametrically opposite directions. In Fig. 4 they follow a downward trend indicating a decrease in death age with increasing litter number, while in Fig. 5 the death age if anything increases with increasing litter number. As the probits of the incidence give a straight line course when plotted against the number of litters and when plotted against age the inference that can be drawn is that the probit procedure indicates the existence of an identical basic mechanism, but does not permit conclusions as to the effect

Fig. 5.—Ordinate : Average age at death in days of RIIIf breeders.
Abscissa : Number of litters.

of litter number and/or to the effect of age upon the incidence of mammary tumours.

SUMMARY

1. 108 $C_3H_f$ and 544 $RIII_f$ breeding females have been observed. The number of litters each female had and her survival age or the date of appearance of a mammary carcinoma have been recorded. There were 28 $C_3H_f$ and 14 $RIII_f$ mammary carcinomas. The incidences of tumours of other sites have been included.

2. It was not found possible to draw any conclusions as to the effect of litter number and/or the effect of age upon the incidence of mammary tumours.

Examinations for cytoplasmic hepatoma inclusions were made by H. M. Laird.

### REFERENCES

Agnew, L. R. C. and Gardner, W. U.—(1952) *Cancer Res.*, **12**, 757.
Andervont, H. B.—(1950) *J. nat. Cancer Inst.*, **11**, 581.
*Idem* and Dunn, T. B.—(1952) *Ibid.*, **13**, 455.
*Idem* and McEleney, W. J.—(1941) *Ibid.*, **1**, 737.
Burns, E. L. and Schenken, J. R. (1943) *Cancer Res.*, **3**, 691.
Clyde, J. D., Law, L. W. and Dunn, T. B.—(1959) *J. nat. Cancer Inst.*, **23**, 717.
Dunn, T. B.—(1953) *Ibid.*, **14**, 1281.—(1959) in ' Physiopathology of Cancer ', edited by Homburger, F. and Fishman, W. H. London (Cassel and Co. Ltd.), 2nd edition, p. 38.
Foulds, L.—(1956) *J. nat. Cancer Inst.*, **17**, 701.
Head, M. A. and Laird, H. M.—(1956) *Rep. Brit. Emp. Cancer Campgn*, **34**, 282.
Heston, W. E.—(1958) *Ann. N.Y. Acad. Sci.*, **71**, 931.
*Idem* and Deringer, M. K.—(1952) *J. nat. Cancer Inst.*, **13**, 167.—(1953) *Proc. Soc. exp. Biol., N.Y.*, **82**, 731.
*Iidem* and Dunn, T. B.—(1956) *Ibid.*, **16**, 1309.
*Iidem* and Levillian, W. D. (1950) *Ibid.*, **10**, 1139.
Hummel, K.—(1958) *Anat. Rec.*, **132**, 281.
Jones, E. E.—(1940) *Amer. J. Cancer*, **39**, 94.
Mühlbock, O.—(1950) *J. nat. Cancer Inst.*, **10**, 1259.
Prehn, R. T., Main, J. M. and Schneiderman, M.—(1954) *Ibid.*, **14**, 895.
Pullinger, B. D.—(1952*a*) *Brit. J. Cancer*, **6**, 69.—(1952*b*) *Ibid.*, **6**, 78.—(1955) *Ibid.*, **9**, 613.—(1959) *Ibid.*, **13**, 99.
Pybus, F. C. and Miller, E. M.—(1940) *Amer. J. Cancer*, **40**, 47.
Shimkin, M. B.—(1945) in a Symposium on Mammary Tumors in Mice. *Amer. Ass. Advanc. Sci., Wash.*, p. 85.
Simonds, J. P. (1925) *J. Cancer Res.*, **9**, 329.
van Rÿssel, T. G. and Mühlbock, O.—(1955) *J. nat. Cancer Inst.*, **16**, 659.
Whitehead, R.—(1932) *J. Path. Bact.*, **35**, 415.
Woolley, G. and Little, C. C.—(1945) *Cancer Res.*, **5**, 193

### Addendum.

Thanks to the kind collaboration of Professor M. G. P. Stoker and Dr. M. Sussman of the M.R.C. Virology Unit, Glasgow, sample sera from $RIII_f$ and $C_3H_f$ mice have been tested for polyoma antibody. Hæmagglutination inhibition titres over 1/320 were found in both strains.